# RIS-UNet: A Multi-Level Hierarchical Framework for Liver Tumor Segmentation in CT Images

**DOI:** 10.3390/e27070735

**Published:** 2025-07-09

**Authors:** Yuchai Wan, Lili Zhang, Murong Wang

**Affiliations:** 1 Beijing Key Laboratory of Big Data Technology for Food Safety, Beijing Technology and Business University, Beijing 100048, China; zhanglili_955@163.com; 2 Femtosecond Applications and Research, Science and Technology Innovation Base, No. 80, Lanyue Road, Science City, High-Tech Industrial Development Zone, Huangpu District, Guangzhou 510700, China; murongwang@outlook.com

**Keywords:** liver tumor segmentation, multi-level, deep learning, CT images

## Abstract

The deep learning-based analysis of liver CT images is expected to provide assistance for clinicians in the diagnostic decision-making process. However, the accuracy of existing methods still falls short of clinical requirements and needs to be further improved. Therefore, in this work, we propose a novel multi-level hierarchical framework for liver tumor segmentation. In the first level, we integrate inter-slice spatial information by a 2.5D network to resolve the accuracy–efficiency trade-off inherent in conventional 2D/3D segmentation strategies for liver tumor segmentation. Then, the second level extracts the inner-slice global and local features for enhancing feature representation. We propose the Res-Inception-SE Block, which combines residual connections, multi-scale Inception modules, and squeeze-excitation attention to capture comprehensive global and local features. Furthermore, we design a hybrid loss function combining Binary Cross Entropy (BCE) and Dice loss to solve the category imbalance problem and accelerate convergence. Extensive experiments on the LiTS17 dataset demonstrate the effectiveness of our method on accuracy, efficiency, and visual results for liver tumor segmentation.

## 1. Introduction

In recent years, the incidence rate of hepatocellular carcinoma has increased gradually. According to the national cancer statistics in 2022 [1], the incidence rate of liver cancer in China ranked fourth in the incidence rate of malignant tumors in 2016, while the mortality rate of liver cancer ranks second. Therefore, the early diagnosis and treatment of liver tumors are crucial.

Computed Tomography (CT), as one of the main methods for liver cancer diagnosis, is widely utilized. In clinical settings, taking a CT scan will produce a 3D image volume, containing hundreds of 2D CT slices. The 3D volume visually presents the spatial structure of the liver and tumor, enabling doctors to observe the morphology of the liver and tumor from different angles. Lining out the tumor region is important in clinical practice. However, at present, the liver and tumor regions in CT image are usually manually labeled slice by slice by doctors, which is both time-consuming and susceptible to the operator’s subjective influence. To alleviate doctors’ workload and overcome the limitations of manual annotation, an increasing number of studies are exploring automatic liver tumor segmentation methods.

Recently, deep learning-based methods have made some significant progress in multiple domains. Numerous Convolutional Neural Network (CNN)-based architectures [2], such as U-Net [3], U-Net++ [4], and AttentionU-Net [5], have been applied to tissue segmentation, lesion recognition, etc. Specifically, dedicated network structures, like H-DenseUnet [6], have been developed for liver lesions. These networks optimize medical image segmentation through specific designs tailored to liver tissue structure and lesion characteristics, achieving remarkable improvements in segmentation accuracy for liver lesions.

**Figure 1:**
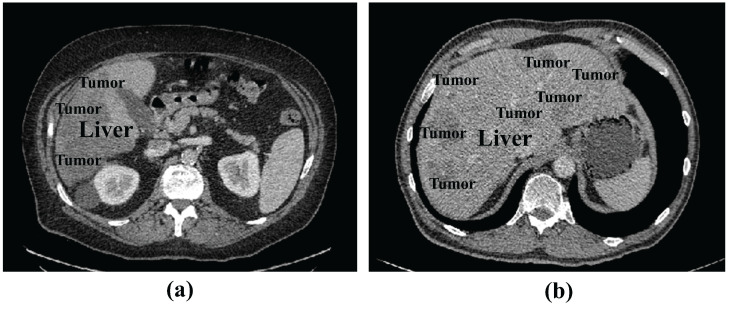
Examples of liver CT images: (**a**) tumors located around the liver boundary (**b**) small and low-contrast liver tumor.

However, limitation still exists for CNN-based techniques to meet the high precision requirement for clinical applications. Figure 1 illustrates the examples of liver and tumor CT images. We can see that, in abdominal CT images, the liver, as well as the lesion region, have low contrast with the surrounding tissues, and a single CT slice provides limited information. The accuracy of 2D analysis methods based on a single CT slice is not satisfactory, although they can achieve high segmentation efficiency. Segmentation methods based on 3D images can fully utilize spatial information to enhance segmentation accuracy, but the parameter of 3D deep learning models are substantially higher, requiring higher computational resources; 2D and 3D segmentation methods are unable to strike a balance between efficiency and accuracy. Furthermore, liver tumors in CT images have different sizes and locations, and their visual features need to be fully extracted to be able to adapt to the changes in tumor size and make the segmentation results closer to the true ground truth.

To solve these problems, we propose a multi-level hierarchical framework for liver tumor segmentation, which designs a Res-Inception-SE module and combines it with the U-Net architecture, named as RIS-UNet. The model adopts a two-level strategy. The first level designs a 2.5D network for inter-slice spatial information integration, which significantly enhances the 3D spatial context information modeling capability while maintaining high computational efficiency. The second level performs inner-slice feature extraction to capture global and local features in slices.

Our main contributions are as follows:

(1) We propose a multi-level hierarchical framework for liver tumor CT segmentation, considering both inter-slice and inner-slice information.
(2) We design a 2.5D segmentation structure that introduces a continuous multi-slice input strategy (*n* = 1/3/5/7) to address inter-slice feature learning limitations. This framework achieves a balance between segmentation accuracy and efficiency.
(3) We propose a Res-Inception-SE-Block, aiming to capture more inner-slice global and local feature information. The module adopts the collaborative design of multi-scale convolution decomposition, residual connection, and a channel attention mechanism to achieve more complete tumor region segmentation and accurately identify small tumors that are difficult to segment.
(4) We design a hybrid loss function combining Binary Cross Entropy (BCE) and Dice loss to solve the category imbalance problem and achieve fast convergence.

The rest of the paper is organized as follows: Section 2 presents related work; Section 3 describes the proposed method in detail; Section 4 provides the experimental configurations, results, and in Section 5 we summarize the study and present future perspectives.

## 2. Related Work

### 2.1. Medical Image Segmentation Methods

In medical image analysis, the purpose of lesion segmentation is to distinguish abnormal and normal regions, which assists in disease diagnosis and surgical resection tasks. Early medical image segmentation techniques were mainly based on traditional machine learning methods, including support vector machines [7,8], random forests [9,10], the K-nearest neighbor algorithm [11], and decision trees [12]. These methods utilize manually extracted features such as texture, shape, and edge information to distinguish different regions in an image. In addition to these supervised learning methods, there are also unsupervised learning methods such as fuzzy C-means clustering [13] and K-means clustering [14], which assign image pixels to different clusters in order to achieve regional segmentation of an image. Graph-based segmentation methods [15] were also used in early medical image segmentation, which find the optimal segmentation by converting an image into a graph and defining an energy function on the graph by minimizing this energy function. Although these traditional methods worked well, they usually rely on expert experience to select and adjust features and algorithm parameters, which may limit their efficiency and automation when dealing with complex medical images.

The rise of deep learning techniques has made the field of medical image segmentation more automated and efficient. Moreover, some important architectural innovations have emerged in CNNs. Szegedy et al. [16] proposed the Inception architecture (including variants V1–V4), featuring multi-scale convolutional filters within modules to efficiently capture features at different resolutions. Long et al. [17] introduced Fully Convolutional Networks (FCNs), which employ convolutional layers to achieve end-to-end learning, thus transforming the segmentation task into a pixel-by-pixel classification problem. Christ et al. [18] introduced a cascade network based on FCNs for the segmentation of livers and tumors, which starts from the tumor region in a stepwise manner, and finally refined the results using conditional random fields.

Subsequently, Ronneberger et al. [3] proposed U-Net, which is a U-shaped network designed for biomedical image segmentation, adopting an “encoder–decoder” architecture. The symmetric encoding and decoding pathways of U-Net provide an efficient structural framework for feature abstraction and recovery, while its hopping connections effectively preserve image details. The design is especially suitable for medical images with limited data, and it has significantly influenced the field. Zhou et al. [4] considered that features at different depths have different roles and extended the hopping connections between the deep and shallow network layers into a combination of long connections between the deep and shallow layers and short connections between the neighboring layers, which is named UNet++. Huang et al. [19] introduced the UNet3+ model, which introduces a full-scale jump connection strategy, which effectively fuses shallow semantic information with deeper advanced features in different scale feature maps. Qian et al. [20] introduced UNet# based on these UNet-like networks. This model combines dense and full-size jump connections to effectively integrate the semantics of different scales of feature maps. Further, Wang et al. [21] proposed a dual-branch dynamic hierarchical U-Net (D2HU-Net) with multi-layer spatial fusion attention, in which the shallow decoding path provides guidance for the deep decoding path to achieve more advanced results.

Despite the significant advancements achieved by CNN-based methods in the field of medical image segmentation, especially in feature extraction and local information capture, these approaches exhibit limitations in capturing information due to their inherent local-awareness properties. For this question, the Transformer model [22,23] is able to effectively capture long-range dependencies and provide more detailed and comprehensive image feature representations due to its strong information modeling capability and self-attention mechanism, thus demonstrating better segmentation performance in some scenarios compared to traditional CNN methods. For example, MISSFormer [24] replaces the U-shaped codec stage with a pure Transformer module and incorporates the feedforward network of the underlying Transformer into the residual convolutional network idea, while utilizing the module to increase the richness of the jump-connected supplementary features. MISSFormer shows some advancement in abdominal organ segmentation such as liver, while Swin-UNet [25] incorporates the Swin Transformer [26] module into U-Net and introduces a segmentation model with pure Transformer architecture, which is effective in abdominal organ segmentation. The Swin Transformer-based UperNet (Swin-TransUper) [27] introduced the Swin Pyramid Pooling Module (SPPM) to extract the deepest-level features of the image, enabling the model to fully capture and utilize semantic information. In addition to the pure Transformer architecture, many current works incorporate the inductive bias of CNNs to design hybrid models. TransUnet [28] first extracts the low-level features using CNNs, and then models the global interactions through a Transformer, setting a new record in CT organ segmentation tasks such as the liver. Furthermore, Cai et al. [29] proposed a 3D variants of Swin-UNet, which adopted the parallel architecture of a convolution and visual Transformer, so that all layers could adequately learn the global and local dependency information. Inspired by Transformer, Roy et al. [30] introduced a large convolutional kernel segmentation network called MedNeXt, which aims to maintain the semantic richness between different scales through ConvNeXt upsampling and downsampling modules with a residual mechanism. Yang et al. [31] introduced a D-Net model for 3D volume medical image segmentation. In this work, they designed a dynamic large kernel module and a dynamic feature fusion module, which are embedded into a hierarchical Transformer architecture. D-Net can effectively leverage multi-scale receptive fields and adaptively utilize global context information. Perera et al. [32] introduced a lightweight SegFormer3D that uses a hierarchical Transformer to calculate attention across multi-scale volume features. Furthermore, a full MLP decoder is designed to aggregate local and global attention features to produce highly accurate segment masks.

### 2.2. Network Architectures for CT Image Segmentation

Liver CT scans can generate 3D volume images. Currently, deep learning-based liver tumor segmentation architectures can be divided into two classes: 2D methods and 3D methods.

Most of the current liver tumor methods use a 2D neural network framework, i.e., a 2D CT slice is used as the model input. For example, Seo et al. [33] optimized the jump connections of the U-Net model by introducing a residual path. It produces improved segmentation results for liver and tumor regions when dealing with ambiguous segmentation boundaries and small objects. Almotairi et al. [34] improved SegNet [35] by retaining only the maximal pooling indices in the feature maps instead of the complete feature maps, enabling more accurate tumor segmentation. F. Lyu et al. [36] introduced an approach using the Couinaud fragment annotation to train networks for liver tumor segmentation, which not only has a higher segmentation accuracy but also requires significantly less annotation work. Sun et al. [37] proposed RHEU-Net, a multi-scale liver tumor segmentation method based on residual modules and hybrid attention mechanisms, significantly improving segmentation accuracy. Liu et al. [38] proposed a novel network named SEU2-Net by introducing the attention mechanism into U2-Net for accurate and automatic liver occupying lesion segmentation. These 2D networks show better performance, but since they analyzed based on only one CT slice, without considering the information of its spatially neighboring slices, the accuracy is difficult to further improved.

To effectively utilize the spatial information between the slices of a 3D image, Ciek et al. [39] introduced 3D U-Net, which performs feature extraction operations by 3D convolution to better capture spatial information in 3D images. Furthermore, Song et al. [40] enhanced 3D U-Net and proposed the ELANRes-MSCA-UNet model. This model significantly improved feature extraction and boundary optimization, particularly excelling in segmenting small targets. Li et al. [6] designed H-DenseU-Net, which combines 2D and 3D DenseNet blocks to efficiently extract and fuse inter- and intra-slice features for further better performance. Subsequently, Wang et al. [41] designed a 3D network MSA-Net, which directs the model to focus on important features through attention mechanism, while a fusion module is used to capture multi-scale feature. These 3D methods can segment along the axial direction to utilize the spatial information. However, they have substantially higher parameter amount and require more hardware computational power and longer inference time.

To balance the problems of the accuracy and efficiency of 2D and 3D strategies, researchers have proposed to fuse these two strategies, namely the 2.5D strategy. Vu et al. [42] superimposed neighboring slices as input data and fed them into a 2D network to carry out model training, and segmentation was performed based on the obtained 2D feature maps. Similarly, Lv et al. [43] introduced a new 2.5D framework RIU-Net, using lightweight RI modules for quick and precise segmentation of liver tumor from CT. Xia et al. [44] proposed a multi-path Transformer fusion network (MTr-Net) that combines Z-axis information from 3D networks to address tumor boundary deformation, while balancing contextual residual information to further refine the segmentation results of organs and tumors. Fırat et al. [45] proposed a new hybrid AIM-UNet model, which combines U-Net and Inception V3 architecture for liver and tumor segmentation. Furthermore, more advanced approaches begin to explore the fusion of 2.5D and attention mechanisms to learn the correlation of slices in 2.5D images. For example, Zeng et al. [46] proposed DAL-UNet, a Ladder-ASPP module integrated with a 2.5D dual attention network, which focuses on the target region more efficiently to improve the tumor segmentation accuracy. Sun et al. [47] developed a new 2.5D multi-attention perception-fusion U-Net (MAPFU-Net) for liver segmentation. It is used to reduce the gap between the decoder and the encoder, which produce features for segmentation. The multi-scale features are extracted from CT scan images, which minimizes the computational cost in segmentation.

Although deep learning-based approaches improve performance, some challenges remain. For example, as the small size lesions contain limited information, the image scaling and pooling operations during the training process further reduce the information, leading to the difficulty of recognizing small lesions. Meanwhile, the grayscale similarity and fuzzy tumor boundaries in liver images increase the difficulty of complete large tumor segmentation. In addition, the existing 2.5D segmentation methods only utilize inter-slice spatial information without exploring segmentation effect with different sequence numbers.

## 3. The Proposed Methods

**Figure 2:**
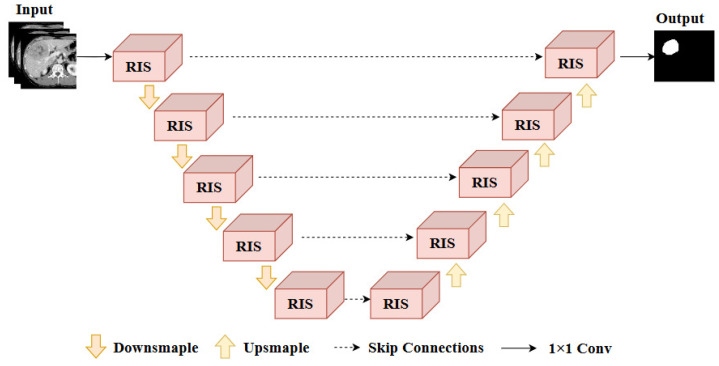
Architecture of the proposed RIS-UNet model.

This paper proposes a RIS-UNet for liver tumor segmentation, the architecture of which is shown in Figure 2. To improve the segmentation accuracy and maintain high efficiency simultaneously, we propose a multi-level hierarchical segmentation architecture. Considering that the 2D deep learning models lack the utilization of inter-slice spatial information and the 3D deep learning models require long inference time, the first level of our method performs inter-slice spatial information fusion and designs the 2.5D network, in which a set of spatial neighboring slices are used as inputs to the model. Next, the second level focuses on the inner-slice global and local feature extraction to gain a comprehensive description of liver and tumors. The proposed RIS-UNet model utilizes the spatial neighboring slices as input, and then employs a five-layer encoder–decoder structure for feature extraction from the input images. In each encoder and decoder module, Res-Inception-SE-Block (RIS) is designed to replace the convolution operation in the original U-Net for local and global information feature extraction. The encoder and decoder are connected by skip connections to obtain richer feature representations and thus better mitigate inner-class differences.

In the encoder, feature dimensionality reduction is conducted by using a maximum pooling operation after each layer of RIS processing. The decoder structure processes the number of channels and gradually restores the resolution of the feature map by up-sampling. After 1 × 1 convolution, we obtain the final output, which is the prediction map. The RIS module is able to capture the multi-scale features of liver tumors, thus enabling the network to focus on the information related to the location of liver tumors for accurate pixel-level segmentation.

### 3.1. Inter-Slice Spatial Information Integration

Traditional 2D convolutional neural networks usually process only a single slice, which limits the model’s utilization of spatial information between adjacent slices. To fully exploit the contextual information in 3D images, we design a 2.5D deep learning model, which employs n consecutive neighboring slices as inputs. This strategy not only improves the model’s representational capability by introducing multi-slice spatial information but also avoids the high computational cost of 3D convolution modeling.

Assume that the original 3D medical image volume is $V\in R^{H\times W\times D}$, where *H* and *W* denote the height and width of the slices, respectively, and *D* is the number of slices. For the *t*-th slice $I_{t}\in R^{H\times W}$, its corresponding multi-slice input $X_{t}$ consists of *n* consecutive slices centered on $I_{t}$, as shown in Equation (1).

(1) $$X_{t}=\left[I_{t-\left\lfloor n/2\right\rfloor },\ldots ,I_{t},\ldots ,I_{t+\left\lfloor n/2\right\rfloor }\right]\in R^{H\times W\times n}$$

where *n* is odd to ensure symmetry. For boundary slices (e.g., $t<\left\lfloor n/2\right\rfloor$ or $t<D-\left\lfloor n/2\right\rfloor$), zero-filling is used to complement the missing slices.

In order to fuse multi-slice information, we modify the deep learning model to fit the multi-slices input. In the first convolutional layer, we extend the convolutional kernel as kernel∈R, where kernel is the spatial dimension (e.g., 3 × 3). The process is shown in Equation (2).

(2) $$Conv_{kernel}\left(X_{t}\right)=\sum_{i=1}^{n}W_{i}*I_{t+i-\left\lfloor n/2\right\rfloor }+b$$

where $W_{i}$ is the weight corresponding to the *i*-th input channel, * denotes the convolution operation, and *b* is the bias term.

In the experimental section, we compare the model performance with different values of $n\in \left\{1,3,5,7\right\}$ and use evaluation metrics to select the most appropriate value of *n*.

### 3.2. Inner-Slice Global and Local Feature Extraction

**Figure 3:**
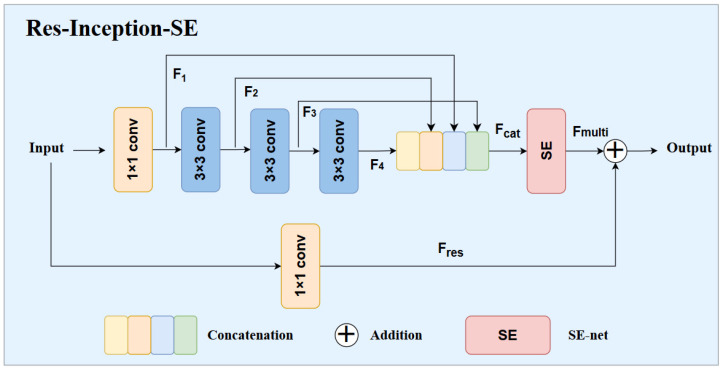
Structure of Res-Inception-SE module.

In the U-Net architecture, each convolution layer consists of two 3 × 3 convolutions. But there are significant differences in the size of liver tumor regions, so the fixed receptive field can only extract local feature information, resulting in insufficient feature capture, low utilization, and limited liver tumor segmentation accuracy. To obtain comprehensive feature integration, inspired by the attention mechanism and the Inception structure [16], we propose a Res-Inception-SE module, which mainly consists of two 1 × 1 convolution blocks, three 3 × 3 convolution blocks, a residual block, and a squeeze-and-excitation block. Thus, the model can capture features of different scales to improve the segmentation accuracy. Figure 3 illustrates the details of the Res-Inception-SE module.

The Res-Inception-SE module captures the feature information of different receptive fields through the strategy of splitting and fusion. In the splitting stage, the module contains four different branches, each of which performs a convolution operation on the feature map using a series of convolution kernels of different sizes for processing the input features at different scales. First, the input image is processed using a 1 × 1 convolution to obtain $F_{1}$. Subsequently, the feature map $F_{1}$ is processed by three series-connected 3 × 3 convolutional blocks. Specifically, $F_{2}$ is obtained by 3 × 3 convolutional processing, which is used to capture local detailed features. Since two 3 × 3 convolutions are equivalent to a 5 × 5 convolution and three 3 × 3 convolutions can replace a 7 × 7 convolution, such an approach can reduce the computational consumption and the number of parameters for applying large-size convolutional layers and obtain comparable results. Thus, the feature map $F_{2}$ continues to be processed by two 3 × 3 convolutional layers to obtain $F_{3}$ to further extract richer image detail information. Similarly, $F_{3}$ undergoes a combination of multiple convolution and pooling operations to obtain $F_{4}$, which helps to capture large sensory fields and global features. In the fusion stage, features $F_{1}$, $F_{2}$, $F_{3}$, and $F_{4}$ from four different scales are concatenated. The calculation process is shown in Equation (3).

(3) $$F_{cat}=Conv_{1\times 1}\left(Concat\left[F_{1},F_{2},F_{3},F_{4}\right]\right)$$

By capturing the multi-scale features, it can help the model to perceive the data features. However, if the features are simply fused, it may bring a significant amount of redundant information. Thus, the attention mechanism is introduced utilizing the SE block to process the new feature maps $F_{cat}$, so as to capture the correlation between neighboring slices and highlight the key informative features in order to reduce the redundancy of features. In the SE block, the fused feature $F_{cat}$ is used as input to deconstruct the spatial features of the element map using global average pooling. Next, the SE operation is applied to the channel dimension of the feature graph for each slice, allowing the model to adaptively explore the importance of the channels to each slice. The channel attention weights are determined by Sigmoid and ReLU functions. Finally, the original feature maps are weighted and multiplied, so that the network focuses on the parts of interest and suppresses irrelevant noise interference.

Different from the traditional multi-scale convolution module, this module performs secondary fusion for the results after SE block processing. Specifically, the $F_{multi}$ are fused and superimposed with the results $F_{res}$ after passing the 1 × 1 convolution by introducing residual joins. The purpose is to further enhance the features in the region of interest, so that the model pays more attention to the image region of interest and improves the model’s ability to perceive image details and important features.

### 3.3. Loss Function

The complexity of medical images far exceeds that of natural images. For instance, in liver CT scans, liver tumors occupy a small proportion of the image with blurred boundaries and highly diverse morphological variations. This may lead to severe class imbalance. Therefore, we employ the Dice loss function, which calculates the similarity between predicted results and ground truth without considering target scale ratios, making it suitable for small targets like tumors.

However, the traditional Dice loss only focuses on training accuracy and presents inherent limitations. For example, when the model makes an error in some pixel predictions, it can lead to drastic gradient variations in predicted targets, making it difficult for its training process to converge.

The Binary Cross Entropy (BCE) loss function is a fundamental objective function in semantic segmentation, which computes pixel-wise classification errors by measuring the divergence between predicted probabilities and ground truth labels.

To fully leverage both loss functions, we define the final loss function as a weighted sum of Dice loss and BCE loss. This combined approach balances model attention across different classes, enhances stability when handling small targets, and improves segmentation performance. Therefore, we use the joint loss function for liver tumor segmentation, which is able to alleviate the problem of category imbalance of the same emphasis on the overall structure of the loss value. The equation of the joint loss function is shown as Equation (4).

(4) $$L=\alpha L_{\mathrm{Dice}}+\beta L_{\mathrm{BCE}}$$

where α and β denote the weight coefficients to balance the proportion of the BCE loss function and the Dice loss function.

## 4. Experiments and Results

### 4.1. Experimental Setup

Dataset: We tested the proposed method using the publicly available LiTS17 dataset containing liver and tumor data. The LiTS17 dataset comprises 201 contrast-enhanced 3D abdominal CT scans, of which 131 cases are allocated for training and 70 cases for testing. For the training set, MICCAI provides manual annotations for liver and tumor regions created by experienced radiologists. The resolution of the dataset is 512 × 512 pixels, with pixel spacing ranging from 0.6 mm × 0.6 mm to 1.0 mm × 1.0 mm.

Since the test samples do not contain annotations, we used LiTS17 training samples for model training. We randomly divided the 131 samples of the LiTS17 training samples into training, validation, and test sets as an 8:2:2 ratio. To prevent overfitting, we implemented data augmentation strategies, including the random cropping of original images combined with vertical and horizontal flipping.

Evaluation Metrics: The commonly used evaluation metrics in medical image segmentation include Dice Coefficient (DPC), Volumetric Overlap Error (VOE), Relative Absolute Volume Difference (RAVD), Average Symmetric Surface Distance (ASSD), and Root Mean Square Symmetric Surface Distance (RMSD). Given the base truth value *A* and prediction mask *B* of the segmentation, DPC is formulated as Equation (5).

(5) $$\mathrm{DPC}\left(A,B\right)=\frac{2|A\cap B|}{\left|A|+|B\right|}$$

The VOE and RAVD measure the overlap between two volumes, which are represented as Equations (6) and (7).

(6) $$\mathrm{VOE}\left(A,B\right)=1-\frac{\left|A\cap B\right|}{\left|A\cup B\right|}$$

(7) $$\mathrm{RAVD}=\left|\frac{\left|B\right|-\left|A\right|}{\left|A\right|}\right|$$

The ASSD and RMSD are applied to evaluate the segmentation boundary and quantify the difference in surface distance between two volumes. ASSD and RMSD can be defined as Equations (8) and (9).

(8) $$\mathrm{ASSD}=\frac{1}{\left|S(A)|+|S(B)\right|}\times \left(\sum_{a\in S(A)}d\left(a,S\left(B\right)\right)+\sum_{b\in S(B)}d\left(b,S\left(A\right)\right)\right)$$

(9) $$\mathrm{RMSD}=\sqrt{\frac{\sum_{a\in S(A)}d{\left(a,S\left(B\right)\right)}^{2}+\sum_{b\in S(B)}d{\left(b,S\left(A\right)\right)}^{2}}{\left|S(A)|+|S(B)\right|}}$$

where S(A) and S(B) denote the surface voxels of *A* and *B*, respectively; d(x,S(A)) is the shortest distance between a voxel *x* and a set of boundary voxels S(A).

In the experimental results, we added up and down arrows next to the evaluation metrics, where the up arrow indicates that higher values of the evaluation metrics represent better segmentation results. The down arrow indicates that lower values represent superior outcome.

Experimental setting: In the training process, we use standard Adam to optimize the objective function. The learning rate was set as 0.003, with a batch size of 8 and 150 iterations. A random seed of 39 was applied. All experiments (including ours and the compared methods) were conducted on a computer equipped with a Nvidia Tesla V100-PCIE GPU and 16GB of RAM.

In the preprocessing stage, we first set the window and level to 40~400 and adjusted the HU value of CT range to [−200, 200]. Then, the image of 512 × 512 size was cropped to 448 × 448 size to remove some of the background. The histogram equalization was performed to enhance the contrast of the image, and then the image pixels were normalized to [0, 1]. Finally, since the model performs the task of liver tumor segmentation, we processed the slices during the training stage by removing the slices without tumors. For each slice, we took *n* consecutive slices centered on it as a group and saved them as an npy file as model input. The size of the npy file was (*n*, 448, 448).

### 4.2. Experimental Results

Number of input slices validation: To determine the appropriate number of input slices, we conducted comparative experiments with different input slice values for the 2.5D input. Table 1 shows four different experiments using consecutive slice numbers *n* 1, 3, 5, and 7 on the LiTS17 dataset. The following can be seen: (1) The best experimental results were obtained with an input of three consecutive slices. (2) Increasing the slice number beyond three provided no performance improvement, with a significant performance degradation observed when using seven slices. A possible explanation is that non-adjacent slices containing less relevant information might misguide the segmentation of the central slice. Furthermore, larger slice values increase computational costs. Based on these findings, the number of slices *n* of three is the optimal configuration. Consequently, all subsequent experiments in this study utilize three input slices as the standard implementation.

Comparison with state-of-the-art methods: To evaluate the segmentation performance of the proposed RIS-UNet model, we conducted a comparative analysis with the state-of-the-art U-Net family and its variants, including the U-Net [3], UNetPlusPlus [4], AttentionU-Net [5], RIU-Net [43], AIM-UNet [45], DAL-UNet [46], and MAPFUNet [47]. Among these, RIU-Net, AIM-UNet, MAPFUNet, and DAL-UNet are 2.5D models, and the rest are 2D models. Table 2 lists the qualitative comparison results.

From Table 2, the following can be seen: (1) When comparing the performance of 2.5D methods (including RIU-Net, MAPFUNet, DAL-UNet, AIM-UNet, and RIS-UNet) with conventional 2D methods (U-Net, U-NetPlusPlus, and AttentionU-Net), the experimental results demonstrate that the 2.5D strategies achieve significant performance improvements. (2) compared with the U-Net family and its variants, RIS-UNet performs better in segmentation performance. This indicates that the RIS-UNet model design based on U-Net is more effective. By introducing the Res-Inception-SE structure, the model can capture multi-scale feature information during the encoding and decoding stages, which further improves the model performance. (3) The analysis of (1) and (2) shows that our proposed multi-level method brings performance improvement at every level, further highlighting the superiority of this network in terms of segmentation accuracy and spatial overlap.

To further validate the efficacy of the 2.5D strategy, we incorporated the 2D, 2.5D, and 3D approaches into the U-Net family and its variants, and the results are shown in Table 3 and Table 4. The following can be seen: (1) All models with 2.5D inputs improve segmentation accuracy compared to their 2D inputs. In particular, for U-Net with the added attention mechanism, the 2.5D input brings a performance gain of 4.89%. (2) For the U-Net family, 3D inputs generally outperform all other input configurations in segmentation performance, but the training time for the 3D input is considerably higher. (3) For our model, the 3D input does not necessarily improve the DPC coefficients, but there is a large improvement in ASSD and RMSD. ASSD and RMSD are the key metrics indicating the coherence of segmentation results, and we hypothesize that this is due to the fact that 3D input enhances the coherence of segmentation but introduces a significant amount of redundant information in the slices, which affects the segmentation accuracy. (4) By comparing with RIU-Net, AIM-UNet, DAL-UNet, and MAPFUNet, which are specifically designed for liver tumor segmentation, RIS-UNet is more accurate in segmenting liver tumors. Overall, the 2.5D inputs for all models were more accurate than the segmentation of the 2D models, and no much decrease in accuracy than the 3d inputs, while substantially reducing training time.

**Figure 4:**
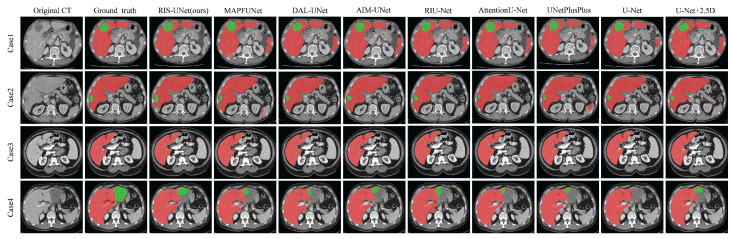
Examples of the segmentation results of different compared models. The red/green part indicates the liver/tumor segmentation result.

In addition, Figure 4 presents the segmentation results of RIS-UNet and the compared models on four clinical cases. The first two columns display original images and corresponding ground truth masks, followed by the segmentation results of different segmentation models.

It can be observed that the other network models encounter over-segmentation or under-segmentation problems. Specifically, the following can be observed: (1) In Case 3, while AttentionU-Net failed to detect small tumor regions, MAPFUNet, DAL-UNet, RIU-Net, AIM-UNet, UNetPlusPlus, and U-Net all managed to segment smaller tumors, but only partially detected them. In contrast, our RIS-UNet accurately identified all small tumors and achieved results that were more in line with the gold standard. (2) In both Case 1 and Case 2, the U-Net family and its variants incorrectly identified the background region as tumors, whereas the improved models based on U-Net all achieved relatively better segmentation. (3) Case 4 is a challenging case due to the tumor’s location at the liver border, where low contrast exists between the liver parenchyma and surrounding tissues. In this case, all models failed to achieve successful tumor segmentation: only partial tumor regions were identified, and some models (U-Net) completely missed the tumor. Compared to other models, our framework performed slightly better in segmentation in the failure cases. (4) When the RIS-UNet model is compared with the model without the RIS module (U-Net+2.5D), we find that the RIS-UNet model with the addition of the RIS module not only segments larger tumor regions more completely, but also detects smaller tumor regions effectively, and the boundary of the segmented lesion is relatively smooth. This indicates that, due to the use of the RIS module in combination with the residual module, multi-scale convolution, and SE module, the key channel feature information is effectively captured while expanding the receptive field, avoiding the information redundancy, effectively capturing small tumor features and improving the tumor detection accuracy. In addition, in comparison with the AIM-UNet model, which is specifically improved for the Inception structure, RIS-UNet also shows better performance, further proving the effectiveness of the RIS module. The above experimental results show that the outstanding performance of RIS-UNet in medical image segmentation tasks.

Table 5 shows the DPC coefficients, parameters, and operating memory during inference for different networks. We can see the following: (1) Adopting 2.5D inputs increases GPU memory requirements but improves segmentation performance compared to 2D input. (2) The proposed RIS-UNet network requires more configurable network parameters. However, such a cost is still reasonable considering the increase in model accuracy.

Analysis of the Res-Inception-SE module: To verify the effectiveness of the Res-Inception-SE module, We conducted experiments on the LiTS17 dataset by replacing the convolutional sequences in the proposed RIS-UNet model with Inception V1, Inception V2, Inception V3, and Inception V4, respectively. To ensure fairness, all the experimental settings are the same except that the convolutional sequences are different. The quantitative results are shown in Table 6. Table 6 shows that the model performance gradually improves with the upgrade of the Inception structure. Specifically, RIS-UNet achieves the best performance in all five metrics compared to other models.

**Figure 5:**
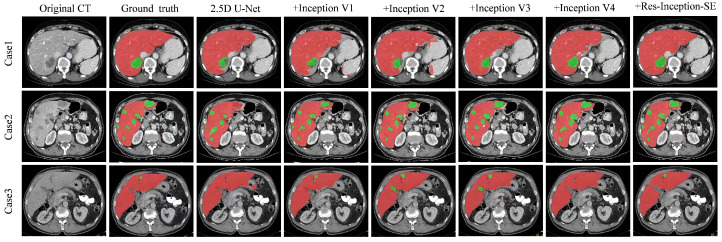
Segmentation results of the different models on the LiTS17 database. The red/green part indicates the liver/tumor segmentation result.

Figure 5 shows the visualization results of the experiment, which is a comparison experiment between our model and replacing the convolutional sequences with a different version of the Inception module. We selected three representative tumors for visualization and comparison: tumors located at the liver boundary, multiple tumors with different shape and location, and small tumors.

From Figure 5, we can see the following: (1) When dealing with the tumor located at the liver boundary in Case 1, the models all show some under-segmentation/over-segmentation errors, and the segmentation results of our model (+Res-Inception-SE) as well as the model with Inception-v4 added are closer to the real boundary of the tumor. (2) The tumor locations in Case 2 are varied in size, and in addition to the large tumor in the upper right, Case 2 contains multiple tiny tumors. It can be seen that U-Net successfully segments some tiny tumors but fails to successfully identify the the large tumor in the upper right. In contrast, our model (+Res-Inception-SE) comprehensively identifies and segments all the tumors in the liver. (3) When dealing with small tumors, other models produced additional or missed small tumors, and even some models did not identify the small tumors. In contrast, our proposed method (+Res-Inception-SE) segmented the small tumor region with high accuracy. Overall, our model has more accurate boundary segmentation for large tumors and can also handle small tumors with diverse shapes and locations.

Ablation Experiments: To validate the effectiveness of our proposed RIS-UNet model, we performed ablation experiments on the LiTS17 dataset. Built upon the baseline U-Net architecture (Base), RIS-UNet combines the 2.5D method, SE block, Res-Inception-SE module, and the hybrid loss function. We progressively incorporated each component to evaluate their contributions to liver tumor segmentation, with experimental results detailed in Table 7.

From Table 7, the following can be seen: (1) The Base (U-Net) achieves a Dice coefficient of 66.05%. With the addition of the 2.5D method and the Res-Inception-SE (RIS) module, the Dice coefficient increases by 2.17% and 7.17%, respectively. When reaching its optimal configuration as the complete RIS-UNet, the model attains maximum segmentation performance with a Dice coefficient of 76.84%. These experimental findings show the effectiveness of these proposed modules and confirm the superior ability of RIS-UNet in segmenting the liver region. (2) Specifically, the comparison of the results of “Base+2.5D” and “RIS-UNet” (RIS-UNet is a RIS model added to the “base+2.5D” model), we can find that the performance of the model with the addition of the RIS module is greatly improved, with an increase of +7.62% in DPC, +8.69% in VOE, +11.74% in RAVD, +1.94% in ASSD, and +1.79% in RMSD. This significant advancement is due to the introduced attention mechanism and residual connections. The channel attention mechanism enables precise focus on crucial segmentation features, while the residual connections complement hierarchical detail outputs. These mechanisms improve segmentation accuracy and operational efficiency by collectively increasing the model’s ability to identify and extract essential information.

## 5. Conclusions

In this paper, we presented a multi-level framework for liver tumor segmentation. In the first level, we design a 2.5D deep learning model using neighboring slices as inputs to balance the segmentation accuracy and computational efficiency between 2D and 3D models. The inter-slice spatial information is utilized comprehensively through the 2.5D model. In the second level, we proposed a Res-Inception-SE module to extract inner-slice global and local features to improve segmentation accuracy. The module utilizes decomposition and combination strategies of convolutions to reduce network parameters while fully leveraging the characteristics of multiple convolutional kernels. At the same time, we introduce residual connections to combine the original features and avoid overfitting. In addition, a hybrid loss strategy combining BCE and Dice losses was designed, to accelerate model convergence and solve the category imbalance problem. Experiments on the LiTS17 dataset have demonstrated that the network can learn more spatial information and global feature information with high segmentation accuracy. When dealing with challenging cases such as livers with small tumors and the boundary segmentation of large tumors, our proposed framework can obtain better results.

While our RIS-UNet framework achieves encouraging results, there are still limitations: the 2.5D design reduces the computational burden of 3D convolution, but the multi-slice inputs still require a higher GPU memory consumption compared to single-slice 2D models, especially when n is large (e.g., *n* = 7). Therefore, future work could explore dynamic slice selection mechanisms to adaptively identify crucial slices. Our framework was experimented on liver CT and could be generalized to other segmentation tasks in the future.

## Figures and Tables

**Table 1:** Results of tests with different slice values.

Number of Slices	DPC (%)↑	VOE (%)↓	RAVD (%)↓	ASSD (mm)↓	RMSD (mm)↓
1	73.22	41.19	25.06	4.01	8.55
3	76.84	36.26	21.38	4.19	10.03
5	74.98	39.29	29.26	4.99	10.36
7	70.43	44.85	33.84	6.06	12.18

**Table 2:** Comparison of five evaluation metrics of our method and the compared methods.

Methods	DPC (%)↑	VOE (%)↓	RAVD (%)↓	ASSD (mm)↓	RMSD (mm)↓
U-Net [3]	66.05	47.52	32.36	5.46	10.79
U-NetPlusPlus [4]	66.08	47.98	33.78	7.21	13.78
AttentionU-Net [5]	66.58	47.32	30.21	6.62	13.63
RIU-Net [43]	70.37	43.19	30.59	6.65	13.04
AIM-UNet [45]	72.51	40.77	24.46	5.14	10.73
DAL-UNet [46]	71.26	43.60	23.43	5.46	10.36
MAPFUNet [47]	73.56	40.52	28.44	5.80	12.74
RIS-UNet (Ours)	76.84	36.26	21.38	4.19	10.03

**Table 3:** Comparison of 2D, 2.5D, and 3D input methods.

Methods	DPC (%)↑	VOE (%)↓	RAVD (%)↓	ASSD (mm)↓	RMSD (mm)↓
U-Net+2D	66.05	47.52	32.36	5.46	10.79
U-NetPlusPlus+2D	66.08	47.98	33.78	7.21	13.78
AttentionU-Net+2D	66.58	47.32	30.21	6.62	13.63
RIS-UNet+2D	73.22	41.19	25.06	4.01	8.55
U-Net+2.5D	68.22	44.95	33.12	6.13	11.82
U-NetPlusPlus+2.5D	68.91	44.88	34.37	5.78	12.27
AttentionU-Net+2.5D	71.27	42.08	28.72	4.80	9.94
RIS-UNet+2.5D	76.84	36.26	21.38	4.19	10.03
U-Net+3D	70.25	43.56	31.63	5.07	10.19
U-NetPlusPlus+3D	71.38	42.69	29.23	5.32	11.64
AttentionU-Net+3D	72.31	41.13	26.52	4.97	9.46
RIS-UNet+3D	75.58	38.23	21.92	3.55	8.05

**Table 4:** Comparison of segmentation accuracy and training time for 2D, 2.5D, and 3D input methods.

Methods	DPC (%)↑	Training Time (h)↓
U-Net+2D	66.05	23 h 58 min
U-NetPlusPlus+2D	66.08	24 h 27 min
AttentionU-Net+2D	66.58	26 h 55 min
RIS-UNet+2D	73.22	33 h 52 min
U-Net+2.5D	68.22	28 h 15 min
U-NetPlusPlus+2.5D	68.91	28 h 47 min
AttentionU-Net+2.5D	71.27	30 h 45 min
RIS-UNet+2.5D	76.84	37 h 05 min
U-Net+3D	70.25	104 h 13 min
U-NetPlusPlus+3D	71.38	127 h 17 min
AttentionU-Net+3D	72.31	152 h 26 min
RIS-UNet+3D	75.58	175 h 08 min

**Table 5:** Comparison of DPC, parameters, and memory by different methods.

Methods	DPC (%)↑	Parameters (M)↓	Memory (MiB)↓
U-Net [3]	66.05	8.64	34.64
U-NetPlusPlus [4]	66.08	9.05	35.47
AttentionU-Net [5]	66.58	8.73	35.01
U-Net+2.5D [3]	68.22	8.64	36.17
U-NetPlusPlus+2.5D [4]	68.91	9.05	36.69
AttentionU-Net+2.5D [5]	71.27	8.73	36.55
RIU-Net [43]	70.37	3.70	24.77
AIM-UNet [45]	72.51	18.10	72.72
DAL-UNet [46]	71.26	12.32	51.76
MAPFUNet [47]	73.56	11.72	34.89
RIS-UNet (Ours)	76.84	15.02	69.20

**Table 6:** Comparative ablation results of tumor segmentation on LiTS17 datasets.

Methods	DPC (%)↑	VOE (%)↓	RAVD (%)↓	ASSD (mm)↓	RMSD (mm)↓
2.5D U-Net	68.22	44.95	33.12	6.13	11.82
+Inception V1	70.34	44.25	31.26	6.79	13.89
+Inception V2	71.67	40.93	28.87	4.91	10.50
+Inception V3	72.31	39.90	28.88	4.65	10.40
+Inception V4	73.45	38.85	27.50	4.38	10.11
+Res-Inception-SE	76.84	36.26	21.38	4.19	10.03

**Table 7:** Ablation experiments.

Methods	DPC (%)↑	VOE (%)↓	RAVD (%)↓	ASSD (mm)↓	RMSD (mm)↓
Base	66.05	47.52	32.36	5.46	10.79
Base+2.5D	68.22	44.95	33.12	6.13	11.82
Base+2.5D+SE	69.33	44.63	32.15	4.58	9.50
Base+RIS	73.22	41.19	25.06	4.01	8.55
RIS-UNet	76.84	36.26	21.38	4.19	10.03

## Data Availability

The data is publicly available at: https://competitions.codalab.org/competitions/17094 (accessed on 6 July 2025).
